# Focused Ultrasound for Noninvasive, Focal Pharmacologic Neurointervention

**DOI:** 10.3389/fnins.2020.00675

**Published:** 2020-07-14

**Authors:** Jeffrey B. Wang, Tommaso Di Ianni, Daivik B. Vyas, Zhenbo Huang, Sunmee Park, Niloufar Hosseini-Nassab, Muna Aryal, Raag D. Airan

**Affiliations:** Neuroradiology Division, Department of Radiology, Stanford University, Stanford, CA, United States

**Keywords:** focused ultrasound, drug delivery, neurointervention, neuromodulation, nanotechnology, blood–brain barrier

## Abstract

A long-standing goal of translational neuroscience is the ability to noninvasively deliver therapeutic agents to specific brain regions with high spatiotemporal resolution. Focused ultrasound (FUS) is an emerging technology that can noninvasively deliver energy up the order of 1 kW/cm^2^ with millimeter and millisecond resolution to any point in the human brain with Food and Drug Administration-approved hardware. Although FUS is clinically utilized primarily for focal ablation in conditions such as essential tremor, recent breakthroughs have enabled the use of FUS for drug delivery at lower intensities (i.e., tens of watts per square centimeter) without ablation of the tissue. In this review, we present strategies for image-guided FUS-mediated pharmacologic neurointerventions. First, we discuss blood–brain barrier opening to deliver therapeutic agents of a variety of sizes to the central nervous system. We then describe the use of ultrasound-sensitive nanoparticles to noninvasively deliver small molecules to millimeter-sized structures including superficial cortical regions and deep gray matter regions within the brain without the need for blood–brain barrier opening. We also consider the safety and potential complications of these techniques, with attention to temporal acuity. Finally, we close with a discussion of different methods for mapping the ultrasound field within the brain and describe future avenues of research in ultrasound-targeted drug therapies.

## Introduction

### Focused Ultrasound as a Potential Modality for Noninvasive Neurointervention

Neuropsychiatric diseases have emerged as one of the largest public health threats today, contributing to an estimated 57% of years lived with disability in the United States from 1990 to 2016 (Mokdad et al., 2018). Treatment of these conditions and other brain disorders is limited by several factors. First, the cytoarchitecture and connectivity of brain regions change significantly every few millimeters (Amunts and Zilles, 2015), and many neuropsychiatric disorders are thought to be mediated by a subset of these different brain areas, demanding a need for focal techniques that can target these specific regions. Second, the blood–brain barrier (BBB) limits the passage of many therapeutics of interest to the brain. Finally, because the brain is a sensitive organ that is only directly accessible by procedures requiring general anesthesia and/or craniotomy, routine direct application of drugs to specific brain targets is many times infeasible at the moment.

**FIGURE 1:**
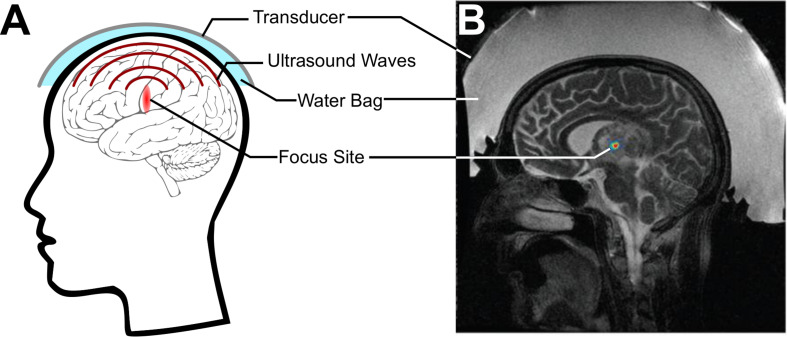
Focused ultrasound (FUS) for noninvasively delivering acoustic energy to the brain. **(A)** Schematic of FUS use. A transducer is coupled to the skin using a water bag and delivers ultrasound waves to a focus within the brain. **(B)** Simulation of energy deposited by a commercial MRI-guided FUS system (Exablate 4000, Insightec, Haifa, Israel) through the skull. Adapted from Vyas et al. (2016). Reprinted with permission from the American Association of Physicists in Medicine.

Focused ultrasound (FUS) is an emerging technology that offers promising strategies to address these issues. Today in the clinic, ultrasound is most often used diagnostically, where a transducer fires ultrasound pulses into the tissue and records returning echoes in order to image the structures within. With the use of transmit focusing (i.e., geometrical and/or electronic), it is possible to noninvasively direct over 1 kW/cm^2^ of acoustic intensity to precisely lesion a specific target site, without substantial energy deposition within the intervening regions between the transducer and the target or regions beyond the focus, an idea initially developed in the 50s by William Fry (Figure 1A; Fry, 1958; Kennedy, 2005). Food and Drug Administration-approved FUS transducers can achieve focusing with millimeter and millisecond resolution anywhere in the brain, whether it be a deep or a cortical structure (Figure 1B; Jolesz, 2009; Liu et al., 2014; Ghanouni et al., 2015).

The predominant clinical use of this technology is for high-intensity FUS operated in a continuous-wave mode to deliver energy to a tightly focused brain region in a completely noninvasive manner. This approach is currently used in the clinic to thermally ablate specific regions of the brain for conditions such as essential tremor and Parkinson’s Disease (Lipsman et al., 2013; Magara et al., 2014; Elias et al., 2016). However, the same FUS systems can be operated in pulsed-wave mode and at lower intensities to enable local drug delivery within the brain. In this review, we will first introduce a number of methods for drug delivery to the brain and then discuss applications of FUS to achieve targeted delivery, namely by temporarily opening the BBB or by directly releasing pharmacologic agents from carrier particles within millimeter-sized structures. We will then close by presenting current techniques that are available for mapping the FUS field within the brain to confirm treatment efficacy and safety, highlighting the need for methods that are well-suited for these low-intensity non-ablative applications.

### Non-ultrasound Methods for Drug Delivery to the Brain

Various methods for delivering drugs directly to the brain have been proposed. One example is osmotic blood–brain barrier opening (BBBO), where a cannula is introduced percutaneously through the arteries to target a cerebral artery. Hyperosmolar mannitol is then injected through the cannula, causing an osmotic shift that disrupts the endothelial cells that partially form the BBB (Burkhardt et al., 2012). Disadvantages of this method include the need for general anesthesia and a high rate of adverse events, including seizures after osmotic BBBO (Marchi et al., 2007). Furthermore, osmotic BBBO covers a wide region of the brain, often opening the BBB across an entire hemisphere in large animals (Joshi et al., 2011), which could be seen as positive or negative depending on the scenario.

Another method being evaluated in small animals currently is laser interstitial thermotherapy (LITT). Here, a fiber optic is introduced into the brain to deliver laser light into a tumor site, improving the blood–tumor barrier permeability to chemotherapeutic agents (Salehi et al., 2019). However, this method requires neurosurgery for a burr-hole craniotomy and insertion of a fiber optic within the brain, limiting the potential indications for this procedure.

Finally, there exist several methods for directly delivering drugs past the BBB. For example, polymer wafers containing the drug of interest can be implanted directly within the brain tissue to slowly release drug into the surrounding cerebrospinal fluid (Brem et al., 1991). One meta-study associated this technique with a 42.7% complication rate, including cerebrospinal fluid leak, infection, cerebral edema, and seizures (Bregy et al., 2013). Another related method is convection-enhanced delivery, where a cannula is stereotactically introduced within the brain and mini-pumps help distribute the drug within the site by convection. Some of these cannulae (up to 68%) can be misplaced, limiting their efficacy (Sampson et al., 2010).

The invasive nature of implanting a foreign object within the brain and the relatively limited efficacy of these methods vs. their complications highlight the urgent need for noninvasive ways to deliver drugs to the brain without requiring general anesthesia or invasive procedures.

## Ultrasound-Based Methods for Drug Delivery to the Brain

### Focused Ultrasound-Mediated Blood–Brain Barrier Opening

#### The Blood–Brain Barrier as a Challenge for Drug Delivery to the Brain

**FIGURE 2:**
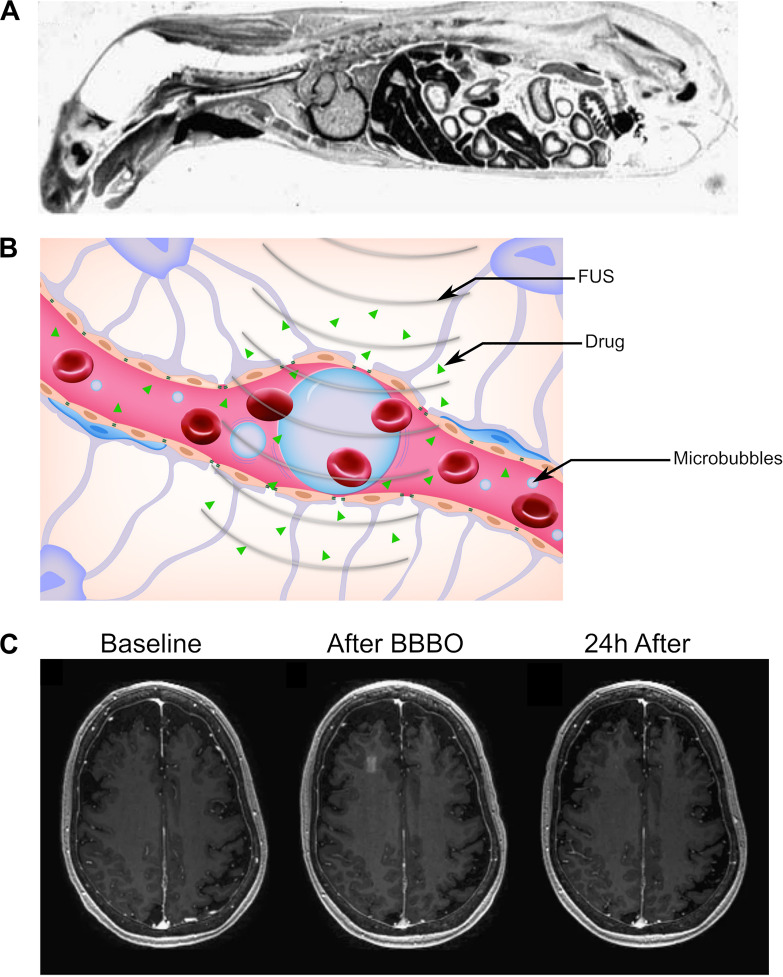
Reversible blood–brain barrier opening (BBBO) with focused ultrasound (FUS). **(A)** Sagittal autoradiography image of a rat following intravenous administration of radiolabeled histamine demonstrates the efficience of exclusion of certain agents from the central nervous system by the blood–brain barrier. Adapted from Pardridge et al. (1986). Reprinted with permission from The American College of Physicians. **(B)** Schematic of BBBO with FUS. Microbubbles (blue) are injected into the bloodstream and are activated by FUS. This causes the spaces between pericytes and astrocytes to open up, enabling delivery of the therapeutic agent (green) past the BBB. **(C)** T1-weighted gadolinium MR images for a patient before (*left*), immediately after (*center*), and 24 h after (*right*) FUS-mediated BBBO. Adapted from Lipsman et al. (2018). Reprinted under Creative Commons License.

At the time of writing, the only ultrasound-mediated method for drug delivery to the brain in clinical trials is FUS-mediated BBBO (Carpentier et al., 2015, 2016; Mainprize et al., 2019). The BBB is formed by a combination of endothelial cells, pericytes, neurons, and astrocytes, all connected with tight junctions and other intercellular connections to form a neurovascular unit that prevents the passage of most small and large molecules, including even water (Abbott et al., 2006). For a more complete description of the BBB (Figure 2A), please refer to recent reviews on the topic by Abbott et al. (2006) and Sweeney et al. (2018b). Physiologically, the BBB serves as a physical barrier that forces most molecular transport through specialized channels, effectively restricting molecular traffic to specific molecules necessary for proper brain function (Abbott et al., 2006). Typically, for passive diffusion across the BBB, a molecule must be both small (<600 Da) and hydrophobic (Norinder and Haeberlein, 2002; Geldenhuys et al., 2015). Otherwise, a compound would need to take advantage of passage through specialized transporters in the BBB to reach the central nervous system (Pardridge, 2005, 2012). Because the BBB is estimated to block 98% of all small-molecule drugs and effectively all foreign large-molecule therapeutics (e.g., monoclonal antibodies), the BBB is considered one of the largest bottlenecks for the development of neuropsychiatric and neuro-oncologic therapies (Pardridge, 2005). Thus, there is a pressing clinical need for methods that can noninvasively, safely, and reversibly open the BBB in and around the target to enable the temporary passage of therapeutic agents to the target. Here, we describe ultrasound-mediated BBBO as a promising technique that meets many of these criteria.

#### Focused Ultrasound and Microbubble-Mediated Blood–Brain Barrier Opening

In its current form, ultrasound-mediated BBBO is achieved by first intravenously injecting microbubbles and shortly thereafter applying short low-pressure (<1 MPa) ultrasound pulses specifically to the target site (Figure 2B; Hynynen et al., 2001). Microbubbles were originally designed to be contrast agents for ultrasound imaging (Chong et al., 2018). They are micrometer-sized particles that consist of a shell (usually either lipid or protein) encapsulating a gaseous core (typically perfluoropropane or sulfur hexafluoride) (Christiansen et al., 1994; Sontum, 2008). Upon sonication with typical BBBO parameters, microbubbles undergo small, stable oscillations, a phenomenon referred to as stable cavitation (Bader and Holland, 2013; Vignon et al., 2013). These oscillations radiate pressure to the surrounding fluid, causing the mechanical formation of pores within the endothelium and opening the tight junctions that form the BBB (Sheikov et al., 2004; Tung et al., 2011). The BBBO can then be visualized with T1-weighted magnetic resonance (MR) imaging after administration of a gadolinium-based contrast agent (Figure 2C).

A wide variety of clinically available and custom microbubbles have been used for BBBO. The most commonly used clinical agents are Optison (Choi et al., 2007), Definity (Baseri et al., 2010), and Sonovue (Fan et al., 2014), whose diameters range from 2.5 to 5 μm. Although these microbubbles might vary in performance at lower pressures (≤0.3 MPa), at higher pressures, these differences are minimized, indicating that beyond some parameter optimization, different microbubbles are functionally equivalent (Wang et al., 2014).

The volume of the affected region depends on the ultrasound focus size and the sonication parameters, highlighting that millimeter-level resolution is achievable with currently clinically available hardware (Ghanouni et al., 2015). After sonication, it has been estimated that the BBB remains open for 24–72 h (Hynynen et al., 2001; Konofagou et al., 2012), with MR-resolved measures of BBBO (e.g., K_trans_) having half-lives of 2–5 h (Park et al., 2012; Chai et al., 2014; Chu et al., 2016).

Agents delivered via ultrasound-mediated BBBO include small-molecule drugs (Hynynen et al., 2001; Park et al., 2012; Aryal et al., 2013, 2014), monoclonal antibodies (Kinoshita et al., 2006a, b), gene delivery vectors (both nonviral and viral) (Hynynen, 2008; Lin et al., 2015; Szablowski et al., 2018), and even stem cells (Burgess et al., 2011). The size of molecules allowed to pass through the pores created with BBBO depends primarily on the peak negative pressure of the ultrasound pulses, with molecules up to 2,000 kDa in size passing through at higher pressures (Chen and Konofagou, 2014). However, BBBO at high enough pressures to allow passage of molecules or cells larger than 500 kDa also led to microhemorrhage on histologic evaluation (Burgess et al., 2011; Chen and Konofagou, 2014). Nonetheless, it is important to note that extravasation of red blood cells after ultrasound-mediated BBBO was not correlated with long-term neural damage (McDannold et al., 2005).

#### Clinical Applications of Blood–Brain Barrier Opening

Currently, the majority of FUS-mediated BBBO trials being conducted in humans are for delivering chemotherapeutic agents to treat brain tumors (Table 1). One approach for clinical BBBO involves the implantation of an unfocused ultrasound transducer within the skull to routinely perform BBBO before chemotherapy administration (Carpentier et al., 2016), whereas another approach uses a noninvasive transducer to perform MR-guided sonication (Mainprize et al., 2019). It is important to note that these studies are often uncontrolled, have less than a dozen subjects, and are primarily powered to evaluate safety and whether BBBO successfully occurred, without assessing the efficacy of ultrasound-mediated BBBO for drug delivery to achieve tumor control.

**TABLE 1:** Clinical trials evaluating ultrasound-mediated blood-brain barrier opening in humans at the time of publication.

Trial number	Start date	Study title	Condition	Location	Status
**Brain cancers**					
NCT02253212	July 2014	Safety of BBB Opening With the SonoCloud (SONOCLOUD)	Glioma or GBM	France	Completed
NCT02343991	October 2014	Blood–Brain Barrier Disruption Using Transcranial MRI-Guided Focused Ultrasound	Primary brain tumors	Canada	Active, not recruiting
NCT03712293	August 2018	ExAblate Blood–Brain Barrier Disruption for Glioblastoma in Patients Undergoing Standard Chemotherapy	GBM	South Korea	Recruiting
NCT03626896	August 2018	Safety of BBB Disruption Using NaviFUS System in Recurrent Glioblastoma Multiforme (GBM) Patients	Glioma or GBM	Taiwan	Completed
NCT03616860	October 2018	Assessment of Safety and Feasibility of ExAblate Blood–Brain Barrier (BBB) Disruption for Treatment of Glioma	GBM	Canada	Recruiting
NCT03714243	October 2018	Blood Brain Barrier Disruption (BBBD) Using MRgFUS in the Treatment of Her2-positive Breast Cancer Brain Metastases (BBBD)	Metastatic HER-2 positive breast cancer	Canada	Recruiting
NCT03744026	February 2019	Safety and Efficacy of Transient Opening of the Blood–Brain Barrier (BBB) With the SonoCloud-9 (SC9-GBM-01)	GBM	France	Recruiting
NCT03551249	March 2019	Assessment of Safety and Feasibility of ExAblate Blood–Brain Barrier (BBB) Disruption	Glioma or GBM	USA	Recruiting
NCT04021420	July 2019	Safety and Efficacy of Sonocloud Device Combined With Nivolumab in Brain Metastases From Patients With Melanoma (SONIMEL01)	Metastatic melanoma	France	Not yet recruiting
NCT04063514	February 2020	The Use of Focused Ultrasound and Microbubble Infusion for Altering Brain Perfusion and the Blood Brain Barrier	Glioma	USA	Not yet recruiting
**Alzheimer’s disease**					
NCT02986932	December 2016	Blood-Brain-Barrier Opening Using Focused Ultrasound With IV Contrast Agents in Patients With Early Alzheimer’s Disease (BBB-Alzheimers)	Alzheimer’s	Canada	Completed
NCT03119961	June 2017	Blood Brain Barrier Opening in Alzheimer’ Disease (BOREAL1)	Alzheimer’s	France	Unknown
NCT03671889	September 2018	ExAblate Blood–Brain Barrier (BBB) Disruption for the Treatment of Alzheimer’s Disease	Alzheimer’s	United States	Recruiting
NCT03739905	December 2018	ExAblate Blood–Brain Barrier Opening for Treatment of Alzheimer’s Disease	Alzheimer’s	Canada	Recruiting
NCT04118764	March 2020	Non-invasive Blood–Brain Barrier Opening in Alzheimer’s Disease Patients Using Focused Ultrasound	Alzheimer’s	USA	Recruiting
**Other**					
NCT03608553	November 2018	A Study to Evaluate Temporary Blood Brain Barrier Disruption in Patients With Parkinson’s Disease Dementia	Parkinson’s disease dementia	Spain	Recruiting
NCT03321487	April 2018	Blood–Brain Barrier Opening Using MR-Guided Focused Ultrasound in Patients With Amyotrophic Lateral Sclerosis	ALS	Canada	Active, not recruiting

*Taken from ClinicalTrials.gov.*

One other exciting application of BBBO is for the direct treatment of Alzheimer’s disease. Preclinical studies have suggested that ultrasound-mediated BBBO could lead to amyloid plaque clearance in preclinical models even without the administration of other therapeutic agents (Jordão et al., 2013; Burgess et al., 2014; Leinenga and Götz, 2015). A recent Phase I safety and feasibility trial in five patients demonstrated no clinically severe adverse events along with no clinically significant worsening in cognitive performance 3 months after BBBO (Lipsman et al., 2018). Further studies are required to establish the mechanism of BBBO for improving Alzheimer’s and to validate and/or refine its use in humans. The use of BBBO to deliver is also actively being investigated in other diseases, such as amyotrophic lateral sclerosis (Abrahao et al., 2019) and Parkinson’s disease (Lin et al., 2016; LeWitt et al., 2019). Again, these preliminary studies were designed to evaluate the safety of BBBO in these patients without actually delivering therapeutic agents through the disrupted BBB. Future trials could be focused on evaluating the efficacy of drug delivery with this method, along with therapeutic efficacy.

In Table 1, we report current and past clinical trials evaluating ultrasound-mediated BBBO in humans (ClinicalTrials.gov, 2020).

#### Safety Considerations of Blood–Brain Barrier Opening

FUS-mediated BBBO is not without risk. The most well-studied adverse effects of FUS-mediated BBBO are the acute complications that arise immediately after sonication, which include microhemorrhage formation (erythrocytic extravasation) and vacuolation of the pericytes and surrounding cells, even at the typical low pressures used (Hynynen et al., 2005; McDannold et al., 2005; Liu et al., 2008; Baseri et al., 2010). At higher pressures, microbubble-enhanced ablation can also occur (McDannold N. J. et al., 2006; McDannold et al., 2013). Hemorrhage can be detected after sonication on MRI using T2^∗^w or susceptibility-weighted imaging (Liu et al., 2008). Nonetheless, as discussed later, there is a pressing need for real-time monitoring to avoid complications during sonication.

**FIGURE 3:**
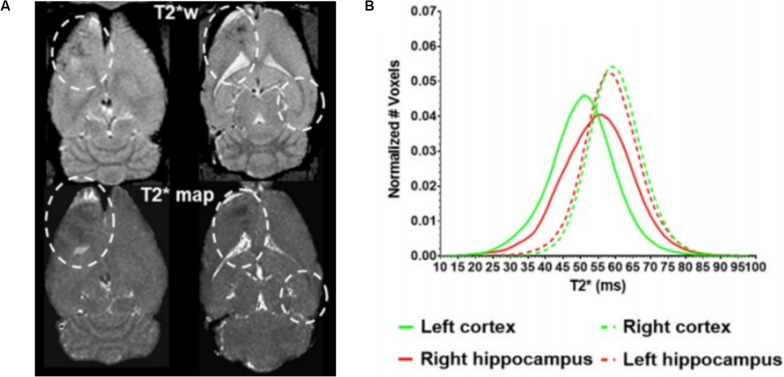
Long-term cortical atrophy after repeated focused ultrasound (FUS)-mediated blood–brain barrier opening sessions. **(A)** Representative T2* images demonstrating long-term effects of 6 weekly BBBO sessions in cortical and deep structures of the brain (white dashed lines). **(B)** Quantification of distribution of T2* times at the targeted sites (solid lines) vs. the contralateral site (dashed lines). Adapted from Kovacs et al. (2018). Reprinted under Creative Commons License.

Beyond these acute effects, the potential for adverse effects of FUS-mediated BBBO in the long term is less well understood or agreed upon. A single session of BBBO was shown to have no effects at least a week beyond treatment, to the resolution of that preclinical analysis (McDannold et al., 2005). Furthermore, there are a number of studies that have performed repeated BBBO sessions for at least 1 month, which found no changes in either MRI, histology, or cognitive testing in nonhuman primates (McDannold et al., 2012; Downs et al., 2015; Horodyckid et al., 2017). However, it is important to note that these BBBO sessions were conducted once every 2 weeks or longer. A contrasting study recently found that repeated weekly BBBO sessions in rats over 6 weeks at the same site led to cortical atrophy, persistent BBB disruption, ventricular size increase, and hyperphosphorylated tau protein buildup at the target site, consistent with neurodegeneration (Figure 3; Kovacs et al., 2018). It is important to note that these effects were not observed with repeated sessions in humans that occurred every 4 weeks with an unfocused transducer, albeit with similar acoustic power (Carpentier et al., 2016). Nonetheless, caution should be taken, especially because histological analysis has revealed sterile inflammation immediately after sonication that persists up to 1 week (Kovacs et al., 2017). Given that neurodegenerative diseases have been closely linked with BBB breakdown (Sweeney et al., 2018a; Nation et al., 2019), further investigation of the long-term effects of repeated BBBO sessions is warranted for the treatment of chronic neurologic conditions without the morbidity and risk–benefit considerations of cancer. Additionally, consideration needs to be given as to how many of these potential effects are due to the permeabilization of the BBB generally vs. the use of ultrasound-induced microbubble cavitation specifically.

### Focused Ultrasound and Nanoparticle-Mediated Drug Uncaging

#### Focal Noninvasive Drug Delivery With Ultrasonic Drug Uncaging

**FIGURE 4:**
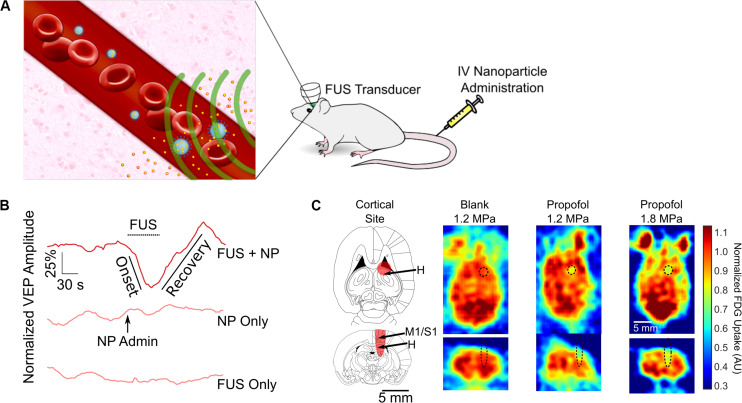
Ultrasonic drug uncaging for spatiotemporally precise neuromodulation. **(A)** Schematic of ultrasonic drug uncaging. Nanoparticles (blue) are administered intravenously, where they are selectively activated by focused ultrasound (FUS) (green). The activated nanoparticles then release their drug (yellow), and the freed drug then diffuses across an intact blood–brain barrier (BBB) into the brain parenchyma (pink). **(B)** Uncaging propofol in the visual cortex silences visually evoked potentials (VEPs), with intensity recovering seconds after ultrasound ceases. **(C)** Fluorodeoxyglucose-positron emission tomography demonstrates that the neuromodulation induced by propofol uncaging is spatially limited to the ultrasound focus (black oval). Adapted from Wang et al. (2018). Reprinted with permission from Elsevier.

As discussed earlier, most work regarding drug delivery in the brain using FUS is centered around BBBO for the delivery of agents that do not normally cross the intact BBB. However, decades of pharmacologic inquiry have yielded libraries of small molecules that are known to normally cross the BBB (Alavijeh et al., 2005; Pardridge, 2012) and are known to have specific action at any of a variety of receptors of importance (Kim et al., 2009; Machado-Vieira et al., 2017). However, these molecules may have adverse effects due to drug action outside the target area in the brain or body, or at the wrong time with respect to the rest of therapy (Haddad and Dursun, 2008). One exciting emerging technology for targeted delivery of drugs that do cross the BBB is the use of ultrasound-sensitive nanoparticles that release their drug payload specifically upon sonication (Airan, 2017). In this application, drug-loaded nanoparticles are intravenously administered, and then, the drug is released (or uncaged) with ultrasound in the intravascular blood volume of the target brain region. The drug then diffuses across the intact BBB into the parenchyma (Figure 4A). These nanoparticles are typically structured as nanoemulsions, with a coat of surfactant such as an amphiphilic block copolymer that encapsulates an ultrasound-sensitive core, typically a liquid perfluorocarbon. The hydrophilic component of the surfactant faces the aqueous medium, whereas the hydrophobic component binds the drug payload and emulsifies the perfluorocarbon droplet. Our group has found that this platform is generalizable to a wide range of hydrophobic drugs, with similar release characteristics and nanoparticle properties regardless of the drug’s identity (Zhong et al., 2019). This criterion allows encapsulation of virtually any drug that is small and hydrophobic, and therefore most drugs of neuropsychiatric interest, as these are the chemical features of drugs that can cross the intact BBB (Norinder and Haeberlein, 2002; Weksler et al., 2005; Geldenhuys et al., 2015).

Notably, the nanoparticles used for ultrasonic drug uncaging can be activated using short low/moderate-intensity ultrasound pulses, namely 1–2 MPa *in situ* at 650 kHz with pulse lengths of 50–100 ms and a pulse repetition frequency of 1 Hz (Airan, 2017; Airan et al., 2017; Wang et al., 2018). These parameters theoretically only lead to a transient 0.1°C temperature increase within the targeted brain region (Wang et al., 2018). This is in contrast to the continuous mode, high-intensity ultrasound protocols required to raise the tissue temperature in order to activate drug release from heat-gated systems like thermosensitive liposomes (Nardecchia et al., 2019). Given the limitations on being able to effectively heat the brain outside the center of the cranium (Odéen et al., 2014) and the risk of heat shock of the brain parenchyma with thermosensitive liposome gating, nanoparticle-mediated ultrasonic drug uncaging is more practically feasible for brain applications.

Most previous work with ultrasound-sensitive nanoparticles have been centered around delivering chemotherapeutics to tumors outside the central nervous system (Rapoport et al., 2009; Fabiilli et al., 2010). In these applications, the nanoparticle uncaging was intended to be completed after the particles were collected within the tumor, taking advantage of the enhanced permeability and retention effect (Rapoport, 2012). In brain applications, because the nanoparticle size (∼300–450 nm) precludes transit across the BBB, the uncaging and delivery occur intravascularly as the uncaged drug diffuses into the brain parenchyma (Figure 4A). Given the types of drugs that are best delivered via ultrasonic drug uncaging, the noninvasive mechanism of delivery, and the high spatiotemporal resolution achieved by FUS, ultrasonic drug uncaging has great potential for neuropsychiatric therapy.

#### Spatiotemporally Precise Neuromodulation With Ultrasonic Drug Uncaging

The use of ultrasonic drug uncaging for spatiotemporally precise neuromodulation was first proposed with the use of nanoparticles loaded with propofol, an anesthetic agent. Preliminary work showed that sonication of propofol-loaded nanoparticles was sufficient to stop seizure activity in the rat, although this work did not fully demonstrate the spatiotemporal resolution of the achieved neuromodulation (Airan et al., 2017). Recently, our group demonstrated by using electrophysiologic recordings and positron emission tomography functional imaging, that the spatiotemporal resolution of neuromodulation is strictly limited by the sonication focus and the kinetics of the uncaged drug, effectively achieving noninvasive neuromodulation with millimeter and second-level resolution for the case of propofol (Figures 4B,C; Wang et al., 2018). With further analysis, we demonstrated that we were able to visualize whole-brain changes that occurred during focal pharmacologic activity at the sonication site, enabling causative mapping of functional networks in the brain with resolutions and a depth of penetration for the causal manipulation that was previously unattainable with noninvasive methods (Wang et al., 2018). As used in combination with positron emission tomography imaging in Wang et al. (2018), ultrasonic drug uncaging could certainly be combined in future efforts with other functional imaging modalities such as functional MRI (Davis et al., 1998), functional ultrasound (Macé et al., 2011), or photoacoustic imaging (Yao et al., 2013). Because ultrasonic drug uncaging does not require any invasive or irreversible procedures such as gene therapy, it is an attractive noninvasive neuromodulation method that could potentially be translated into the clinic. As stated before, ultrasonic drug uncaging is generalizable to excitatory, inhibitory, and neuromodulatory neuropsychiatric drugs (Zhong et al., 2019), enabling selection for the therapeutic effects of these powerful drugs while minimizing off-target effects. Indeed, recently, Lea-Banks et al. (2020) used nanoparticles loaded with pentobarbital to selectively anesthetize part of the rat motor cortex in awake motor tasks.

Other potential uses for ultrasonic drug uncaging include focal treatment of vascular pathologies. Calcium channel blockers such as nicardipine have been encapsulated successfully in these nanoparticles and have been shown to be able to selectively dilate parts of the aorta based on where the uncaging ultrasound transducer was placed (Zhong et al., 2019). Budding applications of this work include the treatment of cerebral vasospasm, a common highly morbid complication of subarachnoid hemorrhage after cerebral aneurysm rupture (Condette-Auliac et al., 2001).

#### Safety Considerations of Ultrasonic Drug Uncaging

It has been hypothesized that ultrasound-sensitive nanoparticles effectively undergo vaporization after exposure to sonication, changing into a gaseous bubble akin to microbubbles used for BBBO (Rapoport, 2012). Theoretically, this would mean that ultrasonic drug uncaging could potentially disrupt the BBB or lead to other forms of cavitation-induced parenchymal injury. However, high-speed microscopy and acoustic recordings by our group have shown that our formulation of these nanoparticles does not undergo vaporization or cavitation during sonication, highlighting their safety in this regard (Zhong et al., 2019). Furthermore, repeated sonication of animals treated with these nanoparticles (upward of two to three times per week for a month) at the same site led to no discernable changes on histology or MRI (Wang et al., 2018).

Our current compositions of these ultrasound-sensitive nanoparticles are made of ingredients that have been approved for human administration by the Food and Drug Administration (Robbin and Eisenfeld, 1998; Makadia and Siegel, 2011). However, a common feature of nanoparticles and microbubbles, in general, is the risk of a hypersensitivity-like reaction upon intravenous administration in humans (Szebeni et al., 2007, 2018; Moghimi, 2018). This reaction is characterized by dyspnea, hypotension, angioedema, and generalized urticaria, similar to anaphylaxic reactions (Moghimi, 2018). It is currently believed that this reaction is not a true anaphylaxis and is mediated through complement and/or macrophage activation and can be controlled through reducing the size of the nanoparticles (which also reduces the sensitivity to ultrasound), changing the shape of the nanoparticle to be less spherical (which has yet to be achieved with ultrasound-sensitive nanoparticles) or slowing the rate of infusion (Moghimi, 2018; Szebeni et al., 2018).

In Table 2, we summarize the features of various methods for drug delivery to the brain, including ultrasound-based and non-ultrasound-based interventions.

**TABLE 2:** Features of different modes of pharmacologic neurointerventions.

Modality	Features
Systemic delivery (IV or oral)	• Noninvasive; Most convenient and conventional mode of delivery • Limited to therapeutics that can cross the BBB (Alavijeh et al., 2005) • High potential for adverse effects due to drug and metabolite action in the body and off-target brain regions (Alavijeh et al., 2005)
Direct Brain Injection and Convection-enhanced delivery	• Highly invasive; potentially injures brain along the cannula path • Directly bypasses the BBB; no limitation on therapeutic agents that could be delivered (Chen et al., 1999) • High spatial resolution, though potentially limited extent of delivery beyond immediate injection zone (Sampson et al., 2010)
Intrathecal administration	• Minimally invasive; requires lumbar or cervical cisternal puncture • Directly bypasses the BBB; no limitation on therapeutic agents that could be delivered (Kim et al., 2016) • Poor penetration into the brain parenchyma (Burch et al., 1988) • Treats the whole cerebrospinal fluid compartment (Burch et al., 1988)
Osmotic BBBO	• Minimally invasive; requires intra-arterial delivery of an osmotic agent and the agent of interest; carries the technical requirements and risks of catheter angiography (Burkhardt et al., 2012) • Temporarily opens the BBB; no definite limitation on therapeutic agents that could be delivered (Burkhardt et al., 2012) • Treats the whole brain region subtended by the artery being infused (Joshi et al., 2011) • Unclear long-term risk profile of increasing BBB permeability, particularly if repeated • Potential for significant acute adverse effects if not well controlled (Marchi et al., 2007)
FUS-mediated BBBO	• Noninvasive; uses ultrasound-induced stable cavitation of intravenously delivered microbubbles (McDannold N. et al., 2006) • Temporarily opens the BBB; no definite limitation on therapeutic agents that could be delivered • High spatial resolution defined by the ultrasound field (Ghanouni et al., 2015) • Unclear long-term risk profile of increasing BBB permeability, particularly if repeated (Kovacs et al., 2018) • Potential for significant acute adverse effects if not well controlled (Baseri et al., 2010)
Ultrasonic drug uncaging	• Noninvasive; uses ultrasound-induced release of drugs from intravenously-administered circulating nanocarriers (Airan, 2017) • Does not disrupt BBB; limited to small hydrophobic therapeutics that can cross the BBB (Wang et al., 2018) • High spatial and temporal resolution defined by the ultrasound field and the action of the drug (Wang et al., 2018) • Potential hypersensitivity-like reaction to the nanoparticles (Moghimi, 2018)

*BBB, blood–brain barrier; BBBO, blood–brain barrier opening; FUS, focused ultrasound.*

## Noninvasively Visualizing the Ultrasound Field Within the Brain for Guidance

The increasing popularity of transcranial FUS applications has propelled the development of noninvasive imaging technologies to fulfill the twofold need for treatment guidance and monitoring.

First, the focusing accuracy must be verified before the treatment. Attenuation and phase distortions are introduced in the propagating ultrasound waves by the acoustically heterogenous skull bone (Clement and Hynynen, 2002), which undermine the focus quality and increase the risk for nonspecific energy deposition in off target brain areas. Moreover, the inherent acoustic propagation properties of the skull are patient-specific and vary significantly between subjects (Vyas et al., 2016). However, if multielement FUS arrays are available, phase correction and adaptive focusing techniques can be applied to restore the desired focusing accuracy, given that the transcranial pressure field can be visualized reliably and noninvasively.

Second, it is paramount to monitor safety parameters and treatment outcomes in real time. To ensure safe and effective ultrasonic exposure levels, real-time monitoring systems have been implemented based on indirect measures of the deposited ultrasonic energy.

In this section, we will review a number of imaging techniques developed to provide focusing quality feedback based on thermal and mechanical acoustic effects in addition to real-time controllers allowing for online safety monitoring. We will limit our analysis to technologies relevant to low-intensity FUS pharmacologic neurointervention.

### Passive Cavitation Monitoring

Originally developed for monitoring high-intensity FUS therapy, passive cavitation detection techniques have been successfully adapted for safety monitoring in applications such as BBBO, which utilize lower ultrasound intensities in combination with intravenously administered gas-filled microbubble contrast agents. When placed within a FUS field, these microbubbles undergo characteristic nonlinear oscillating behaviors (referred to as cavitation), depending upon the ultrasound parameters, including the fundamental frequency of the ultrasound pulse (f_0_), the pulse length, and the applied acoustic pressure. Scattered acoustic emissions generated from cavitation events are detected by passive ultrasound receivers, and the received ultrasonic signals present distinct spectral features. These features carry information on the location, strength, and nature of the cavitation activity. More specifically, cavitation phenomena can be distinguished as inertial or non-inertial. Inertial cavitation is characterized by abrupt particle collapse leading to the production of broadband ultrasonic emissions. This situation is linked to vascular endothelial and parenchymal damage and, therefore, is largely undesired in FUS neurointervention (Hwang et al., 2006). In contrast, non-inertial cavitation is characterized by stable microbubble oscillations induced by relatively weaker ultrasonic energy deposited within the ultrasound focus (Marmottant and Hilgenfeldt, 2003). Such oscillations present harmonic (2*f*_0_, 3*f*_0_, …), subharmonic (1/2*f*_0_), and ultraharmonic (3/2*f*_0_, 5/2*f*_0_, …) spectral components in the detected ultrasonic signals (Sun et al., 2012).

Using passive broadband receivers, usually referred to as passive cavitation detectors, the cavitation activity can be monitored by assessing the spectral content of the received ultrasonic signals. In several studies, the occurrence of broadband emissions from inertial cavitation events was correlated with neurovascular damage and red blood cell extravasation, as confirmed by *post hoc* MRI and histologic findings (Tung et al., 2010; Arvanitis et al., 2012). Conversely, harmonic, subharmonic, or ultraharmonic emissions from non-inertial cavitation could predict effective and reversible BBBO (McDannold N. et al., 2006; Sun et al., 2015). Importantly, multiple real-time safety monitoring systems have been developed based on the online assessment of harmonic, subharmonic, ultraharmonic, and broadband emissions (O’Reilly and Hynynen, 2012; Huang et al., 2017; Sun et al., 2017). This approach was used in recent BBBO clinical trials in amyotrophic lateral sclerosis and Alzheimer’s disease (Lipsman et al., 2018; Abrahao et al., 2019).

**FIGURE 5:**
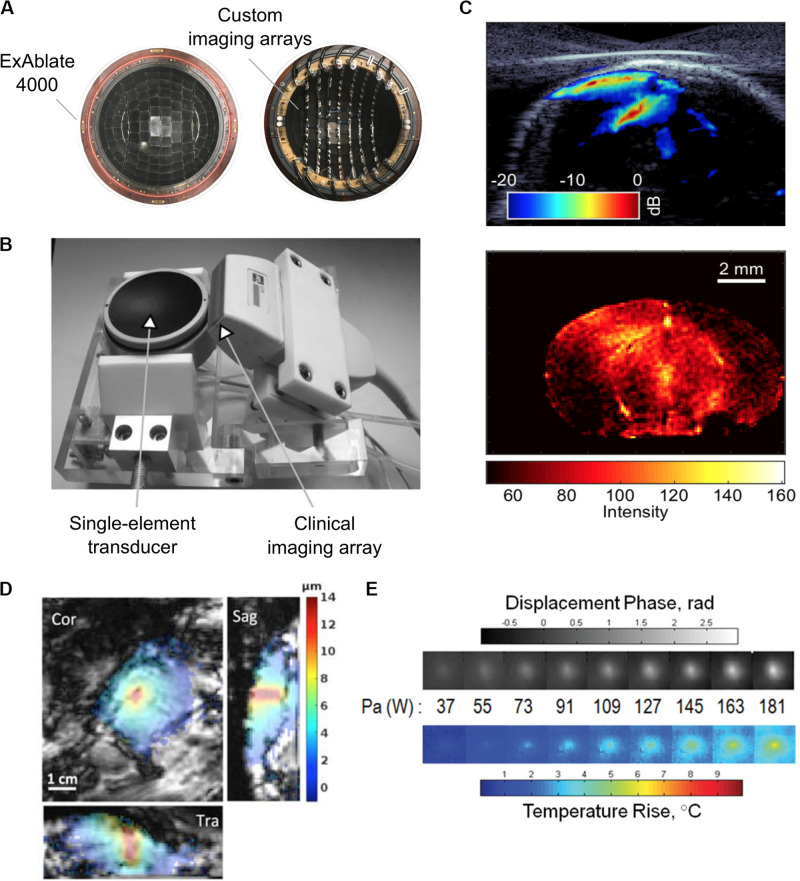
**(A)** Experimental 128-element imaging array (5 MHz) integrated into the 230 kHz hemispherical transducer of an InSightec ExAblate 4000 MRgFUS system for three-dimensional cavitation imaging and skull localization (Crake et al., 2018). © Institute of Physics and Engineering in Medicine. Reproduced by permission of the Institute of Physics Publishing. All rights reserved. **(B)** Conventional ultrasound imaging array (5 MHz) integrated with a single-element therapy transducer (660 kHz) for two-dimensional cavitation imaging (Gateau et al., 2011). Reprinted with permission from the Institute of Electrical and Electronics Engineers. **(C)** (*Top*) Cavitation image overlaid with a B-mode image obtained during an ultrasound-mediated blood–brain barrier opening (BBBO) experiment (Burgess et al., 2018). (*Bottom*) © Institute of Physics and Engineering in Medicine. Reproduced by permission of the Institute of Physics Publishing. All rights reserved. **(D)** Example of magnetic resonance-acoustic radiation force imaging (MR-ARFI) displacement image in *ex vivo* porcine brain. The images indicate the focused ultrasound (FUS) energy distribution (de Bever et al., 2018). Reprinted with permission from John Wiley and Sons. **(E)** Displacement maps and temperature rise measured with a modified MR-ARFI sequence for increasing acoustic power (Kaye et al., 2013). Reprinted with permission from John Wiley and Sons.

Although inexpensive and simple to implement, single detectors can only monitor cavitation activity at the focal region and are unable to spatially resolve different cavitation sources, limiting its ability to directly visualize the total treatment volume (Gyöngy and Coussios, 2010). By integrating multielement receive arrays into the FUS system, on the other hand, spatial maps of cavitation activity can be created from the received broadband signals, allowing for the spatial discrimination of cavitation sources. Based on the multielement passive approach, there have been several implementations of integrated custom and clinical ultrasound imaging arrays with FUS transducers to transcranially monitor BBBO and vascular damage in rodents and in nonhuman primates (Gateau et al., 2011; Arvanitis et al., 2013; Deng et al., 2016; Crake et al., 2018; Figures 5A,B).

In typical BBBO sonication protocols, long sonication pulses make it challenging to use conventional pulse-echo ultrasound imaging techniques for passive cavitation imaging, as the exact time at which a cavitation event occurs is unknown. Numerous passive beamforming algorithms have been developed that rely on the receiver spatial information only to create the cavitation maps (Farny et al., 2009; Salgaonkar et al., 2009; Gyöngy and Coussios, 2010; Haworth et al., 2017). Using beamforming techniques conventionally used in pulse-echo imaging, a recent study used short FUS pulses for BBBO to create cavitation images with improved resolution (Burgess et al., 2018; Figure 5C). Interestingly, another study developed a skull localization and registration routine based entirely on ultrasound data, toward the implementation of an ultrasound-guided FUS platform (Crake et al., 2018). For a comprehensive review of methods for treatment monitoring and control, see Jones and Hynynen (2019).

### Magnetic Resonance Thermometry

MR thermometry offers a possible solution to monitor and guide FUS treatment and to visualize the therapy beam in applications where acoustic feedback from cavitating particles is not available. This technique can noninvasively detect temperature changes in water-containing tissues based on the temperature-dependent proton resonance frequency shift (Chung et al., 1996; McDannold, 2005; Rieke and Pauly, 2008). This approach has proven useful as a pretreatment tool to control ultrasound exposure and to verify the accuracy of the targeting, achieving consistent localization of the sonication profile without apparent tissue damage or BBB disruption, as confirmed by follow-up MRI and histological findings (Hynynen et al., 1997). Importantly, by analyzing the spatial profile of the temperature change, the shape of the ultrasound beam can be inferred, and possible focusing errors or aberrations introduced by the skull can be compensated for before the treatment begins. MR thermometry was combined with passive cavitation monitoring for safety control in recent preclinical and clinical BBBO studies (Huang et al., 2017; Lipsman et al., 2018; Abrahao et al., 2019). Although this technique is routinely used for monitoring of thermal therapies such as low-temperature hyperthermia and ablation (Elias et al., 2013; Lipsman et al., 2013), it has limited effectiveness for real-time safety monitoring in low-power FUS applications due to the low rate of heating involved (< 0.5°C) in these applications.

### Acoustic Radiation Force Imaging

In absorbing tissues, low-power ultrasound pulses exert an acoustic radiation force (ARF) at the focus that moves the tissue away from its resting position along the direction of propagation. McDannold and Maier (2008) demonstrated that the induced longitudinal displacement is linearly proportional to the acoustic power and can be encoded by the phase of the MR signal. Dedicated motion-sensitive MRI sequences have been implemented using displacement-encoding gradients to create displacement maps. These maps provide an indirect measurement of the *in situ* pressure field (Figure 5D). This approach has been tested *in vivo* in rats (Larrat et al., 2010a) and pigs (Hertzberg et al., 2010) and has been optimized to increase the technique sensitivity and to reduce scanning time and heat deposition (Kaye et al., 2011). Currently, the temperature rise induced by magnetic resonance-acoustic radiation force imaging (MR-ARFI) pulse sequences is below 1°C and is considered safe (Table 3 and Figure 5E). However, it is worth specifying that reported ultrasound parameters in MR-ARF imaging studies vary significantly.

**TABLE 3:** Reported sonication parameters for selected publications investigating the use of magnetic resonance-acoustic radiation force imaging (MR-ARFI).

	Acoustic power/pressure/intensity	Frequency	Temperature rise
McDannold and Maier (2008)	<4.1 W (linear regime)	1.63 MHz	0.2–0.5°C (in phantom and kidney *ex vivo*)
Larrat et al. (2010a)	<3.5 MPa	1.5 MHz	Not reported for MR-ARFI
Hertzberg et al. (2010)	Not specified	220 kHz	<1°C (*in vivo;* estimated)
Kaye et al. (2011)	<246 W cm^–2^	550 kHz	No temperature rise detected (*ex vivo*)
Marsac et al. (2012)	172 W cm^–2^	1 MHz	Not reported

Based on MR-ARFI measurements, adaptive focusing techniques have also been developed for correcting phase aberrations to restore the focus sharpness at the target location (Hertzberg et al., 2010; Larrat et al., 2010b; Marsac et al., 2012; Vyas et al., 2012; Kaye and Pauly, 2013). Also, radiation force imaging has been combined with MR thermometry to include safety monitoring capabilities (Kaye and Pauly, 2013; Paquin et al., 2013; de Bever et al., 2018). Currently, the focus intensities needed for MR-ARFI are typically higher than those used for either BBBO or nanoparticle uncaging (Larrat et al., 2010a; Kaye and Pauly, 2013).

## Conclusion

In summary, FUS is an emerging technology that holds great potential for designing noninvasive, targeted pharmacologic neurointerventions with millimeter resolution. We have discussed the use of FUS for opening the BBB to deliver a wide range of large and small molecules, along with the potential safety issues associated with repeated BBBO. We have also reviewed recently developed techniques for directly delivering pharmacologic neuromodulatory agents that normally cross the BBB using ultrasonic drug uncaging. Finally, we provided an overview of the use of passive cavitation mapping, MR thermometry, and MR-ARFI for directly monitoring the sonication field during treatment. It should be noted that these monitoring techniques are mostly limited to use either with microbubbles or with sonication powers higher than those typically used for drug delivery. There is therefore an acute need for the development of more sensitive methods for real-time monitoring of the sonication field during low-intensity applications like ultrasound-mediated drug delivery. Other potential future directions include further investigation of the long-term effects of repeated BBBO and the clinical translation of ultrasonic drug uncaging.

## Author Contributions

JW and TD wrote the initial draft of the manuscript and prepared the figures. All authors contributed to manuscript revisions, read, and approved the submitted version.

## Conflict of Interest

Patent applications have been filed on the nanoparticles described for ultrasonic drug uncaging (17-163 – Provisional application with Stanford University; RDA and PCT/US2017/033226 with Johns Hopkins University; RDA).
